# Downregulation of EphA5 by promoter methylation in human prostate cancer

**DOI:** 10.1186/s12885-015-1025-3

**Published:** 2015-01-22

**Authors:** Shibao Li, Yingfeng Zhu, Chunguang Ma, Zhenhua Qiu, Xinju Zhang, Zhihua Kang, Zhiyuan Wu, Hua Wang, Xiao Xu, Hu Zhang, Guoqiang Ren, Jianmin Tang, Xiangyu Li, Ming Guan

**Affiliations:** 1 Department of Laboratory Medicine, Huashan Hospital, Shanghai Medical School, Fudan University, 12 Central Urumqi Road, Shanghai, 200040 China; 2 Department of Pathology, Huashan Hospital North, Fudan University, Shanghai, China; 3 Department of Urology, Fudan University Shanghai Cancer Center, Shanghai, China; 4 Department of Laboratory Medicine, The people’s hospital of GaoZhou, GaoZhou, China; 5 Central Laboratory, Huashan Hospital, Shanghai Medical School, Fudan University, Shanghai, China; 6 Department of Urology, Huashan Hospital, Fudan University, Shanghai, China

**Keywords:** DNA methylation, EphA5 receptor, Gleason score, Prostate cancer, TNM staging

## Abstract

**Background:**

EphA5 is a member of the Eph/ephrin family and plays a critical role in the regulation of carcinogenesis. A significant reduction of EphA5 transcripts in high-grade prostate cancer tissue was shown using a transcriptomic analysis, compared to the low-grade prostate cancer tissue. As less is known about the mechanism of EphA5 downregulation and the function of EphA5, here we investigated the expression and an epigenetic change of EphA5 in prostate cancer and determined if these findings were correlated with clinicopathologic characteristics of prostate cancer.

**Methods:**

Seven prostate cell lines (RWPE-1, LNCap, LNCap-LN3, CWR22rv-1, PC-3, PC-3M-LN4, and DU145), thirty-nine BPH, twenty-two primary prostate carcinomas, twenty-three paired noncancerous and cancerous prostate tissues were examined via qRT-PCR, methylation-specific PCR, bisulfite sequencing, immunohistochemistry and western blotting. The role of EphA5 in prostate cancer cell migration and invasion was examined by wound healing and transwell assay.

**Results:**

Downregulation or loss of EphA5 mRNA or protein expression was detected in 28 of 45 (62.2%) prostate carcinomas, 2 of 39 (5.1%) hyperplasias, and all 6 prostate cancer cell lines. Methylation of the EphA5 promoter region was present in 32 of 45 (71.1%) carcinoma samples, 3 of 39 (7.7%) hyperplasias, and the 6 prostate cancer cell lines. Among 23 paired prostate carcinoma tissues, 16 tumor samples exhibited the hypermethylation of EphA5, and 15 of these 16 specimens (93.8%) shown the downregulation of EphA5 expression than that of their respectively matched noncancerous samples. Immunostaining analysis demonstrated that the EphA5 protein was absent or down-regulated in 10 of 13 (76.9%) available carcinoma samples, and 8 of these 10 samples (80.0%) exhibited hypermethylation. The frequency of EphA5 methylation was higher in cancer patients with an elevated Gleason score or T3-T4 staging. Following the treatment of 6 prostate cancer cell lines with 5-aza-2′-deoxycytidine, the levels of EphA5 mRNA were significantly increased. Prostate cancer cells invasion and migration were significantly suppressed by ectopic expression of EphA5 in vitro.

**Conclusion:**

Our study provides evidence that EphA5 is a potential target for epigenetic silencing in primary prostate cancer and is a potentially valuable prognosis predictor and thereapeutic marker for prostate cancer.

**Electronic supplementary material:**

The online version of this article (doi:10.1186/s12885-015-1025-3) contains supplementary material, which is available to authorized users.

## Background

Prostate cancer (PCa) is the second most common male malignancy and the sixth leading cause of cancer death in men worldwide [1]. The disease burden is anticipated to grow to 1.7 million new cases and 499,000 deaths by 2030 simply due to the expansion and aging of the global population [2]. Understanding the molecular mechanisms that regulate initiation and progression of PCa is crucial for improving early diagnosis, developing rational therapies, and predicting patient prognosis [3,4]. However, the current information that is available and that can be applied in clinical practice is limited [3,4]. Therefore, the identification of new molecular alterations involved in the initiation and progression of this devastating disease will likely lead to fewer cases of prostate cancer and fewer deaths from the disease.

Eph receptors represent the largest family of receptor tyrosine kinases (RTK) and include 14 human type 1 transmembrane protein members [5,6]. According to sequence homologies and ligand-binding affinities, Eph receptors and their ephrin ligands are divided into the following two subgroups: class A and class B [6]. Eph receptors share a common protein structure, including an extracellular domain (consisting of a globular domain, a cysteine-rich domain, and two fibronectin type III repeats domains), a transmembrane portion and an intracellular domain (consisting of a tyrosine kinase domain, a sterile alpha motif [SAM] and a PDZ binding domain) [7]. The aberrant methylation of CpG island promoter regions of Eph receptor genes is frequently observed during the development of many types of cancers, particularly prostate cancer [8-15].

EphA5, located on chromosome 4q13.1, is a member of the Eph receptor family. In common with other members of the Eph subgroup, EphA5 plays a critical role in the regulation of carcinogenesis and cancer progression [14,15]. Interestingly, a recent transcriptomic analysis revealed that the EphA5 gene is downregulated in radical prostatectomy patients with high grade PCa with a Gleason score of 8,suggesting that EphA5 plays a crucial role in prostate cancer progression [16]. However, the function of EphA5 and its clinical significance in prostate cancer has never been addressed. Here, we demonstrated that EphA5 is frequently downregulated in patients with prostate cancer. Furthermore, we explored the mechanism responsible for the downregulation of EphA5 and investigated its biological function and the association between EphA5 alterations and clinical characteristics of these patients.

## Methods

### Cell culture

The RWPE-1, PC-3 and Du145 cell lines were purchased from the Institute of Biochemistry and Cell Biology, Chinese Academy of Sciences (Shanghai, China). The LNCap, LNCap-LN3, PC-3M-LN4, and CWR22rv-1 cell lines were kindly provided by Dr. Zhang (Biomedical Research Institute, Shenzhen PKU-HKUST Medical Center, Shenzhen, China). All of the cell lines were cultured in RPMI 1640 medium (HyClone, Logan, UT) containing 10% fetal bovine serum, penicillin (100 U/ml) and streptomycin (100 U/ml) in a 5% CO_2_ atmosphere at 37°C.

Cells were seeded at a density of 5 × 10^4^ cells per square centimeter in a 6-well plate. After 36 hours of incubation, fresh culture medium with or without demethylating agent 5-aza-2′-deoxycytidine was added (5 μmol/L, Sigma-Aldrich, USA); cells were then incubated for an additional 48 hours.

### Patients and tissues

All tissue specimens were obtained between March 2013 and December 2013 at the Urology Department of Huashan Hospital (Shanghai, China). Benign prostate hyperplasia (BPH) samples (39) and some of the prostate carcinoma (22) samples were collected from patients undergoing prostate needle biopsies, and 23 paired noncancerous and tumor tissue samples were obtained from patients following radical prostatectomy. Paired normal specimen was obtained from an area that was at least 1cm away from any cancerous tissue and did not contain either cancer cells or premalignant tissue morphologically by histological examination of sequential sections. All of the cancer samples were histologically confirmed to contain greater than 80% tumor cells. Staging was assessed after pathological examination of formalin-fixed specimens according to the 1997 TNM classification system. Written consents were obtained from all subjects and the study protocol was approved by the Ethics Committee of Huashan Hospital. Clinical and biological data from the patients are listed in Table 1.

**Table 1:** **Patient clinical and histological characteristics**

	Prostate cancer (%)	BPH (%)
Case, *n*	45	39
Age (mean ± SD, years)	69.5 ± 10.3	63.6 ± 9.1
TNM		
T_1_	1 (2.2)	
T_2_	12 (26.7)	
T_3_	14 (31.1)	
T_4_	18 (40.0)	
Gleason score		
6-7	22 (48.9)	
8-10	23 (51.1)	
PSA (ng/ml)		
<4.0	2 (4.4)	4 (10.3)
4.0–10.0	7 (15.6)	21 (53.8)
>10.0	36 (80.0)	14 (35.9)
Prostate volume (ml)		
<30	14 (31.1)	8 (20.5)
30–50	17 (37.8)	12 (30.8)
>50	14 (31.1)	19 (48.7)

### Gene expression analysis by real-time PCR

Total RNA was extracted from cells and tissues using the AllPrep DNA/RNA/Protein Mini Kit (Qiagen, Germany), and samples were reverse transcribed using the PrimScriptTM RT Reagent Kit (TaKaRa, China) as described in the manufacturer’s protocol. Real-time PCR to determine EphA5 and GAPDH mRNA levels was performed using the QuantiFast Probe PCR Kit (Qiagen, Germany) according to the manufacturer’s instructions; all analyses were conducted using the ABI Prism 7500 sequence detection system (Applied Biosystems, CA). The relative quantification of EphA5 mRNA levels was performed using the comparative Ct method (2^-△△Ct^ method) with GAPDH as the reference gene. Increases or decreases in mRNA levels of at least two fold were considered to be significant. The primer sequences were as follows: EphA5 forward, 5’-TCTGTGGTACGACACTTGGC-3’; EphA5 reverse, 5’-CTTGCACATGCATTTCCCGA-3’; GAPDH forward, 5’-GAGAAGGCTGGGGCTCATTT-3’; GAPDH reverse, 5’-AGTGATGGCATGGACTGTGG-3’.

### Methylation-specific PCR

Genomic DNA was isolated from cells and tissues using the AllPrep DNA/RNA/Protein Mini Kit (Qiagen, German) and modified using the EZ DNA Methylation-Gold Kit (ZYMO Research Co, Orange, CA) according to the manufacturer’s instructions. To identify aberrant methylation of the EphA5 gene, the modified DNA was amplified using primers specific for the methylated sequence (MSP, forward primer: 5′-ATTGAGTCGTTCGGGATAGC-3′ and reverse primer: 5′-GTCGAAATACAAAATAACAACCGA-3′) and primers specific for the unmethylated sequence (USP, forward primer: 5′-GATTGAGTTGTTTGGGATAGTGG-3′ and reverse primer: 5′-CCATCAAAATACAAAATAACAACCA-3′) using TaKaRa HotStarTaq DNA polymerase [15]. Amplicons were separated on 3% agarose gels and visualized under ultraviolet illumination.

### Bisulfite genomic sequencing

The sodium bisulfite-modified DNA was amplified via PCR using the following primers: Bis-EphA5-F (5′-TGGTTTTTATATTTGGAGGAGT-3′) and Bis-EphA5-R (5′-AAAACCTAAACTCCCAAACC-3′) [15]. PCR products were purified using the QIAquick PCR Purification Kit (Qiagen; Valencia, CA), subcloned into the pMD19-T vector (TaKaRa), transformed into *E. coli* (DH5-alpha) and grown on LB agar plates containing kanamycin with X-gal/IPTG for blue/white selection. To detect the methylation status of the EphA5 promoter, six isolated colonies from each plate were picked, sequenced and analyzed using an ABI 3730 DNA Sequencer (Applied Biosystems).

### Western blot analysis

Total protein was extracted from prostate tissue and cell lines using radioimmunoprecipitation assay (RIPA) buffer, and protein concentrations were determined using the BCA Protein Reagent Kit (Beyotime, China) according to the manufacturer's instructions. Proteins (100 μg) were separated via SDS-PAGE on an 8% gel, transferred to a polyvinylidene fluoride membrane, and incubated with the following antibodies: rabbit anti-EphA5 (1:500, Abcam, CA) and mouse anti-beta-actin (1:1000, Santa Cruz, CA) overnight at 4°C. After washing, the membranes were incubated with HRP-conjugated goat polyclonal secondary antibodies to mouse IgG and rabbit IgG (1:5000, Abcam, CA) for 2 h and visualized with enhanced chemiluminescent substrate (Millipore, CA). Then, immunoreactive bands were quantified using the LAS-3000 system (Fuji Film, Japan).

### Immunohistochemistry (IHC) and Imaging

Tissues were fixed in 4% formalin, embedded in paraffin and sectioned at a thickness of 4 microns. Sections were deparaffinized in several xylene washes and then rehydrated in graded alcohols. The sections were permeabilized in citrate buffer (pH 6.0, Maixin) for 10 min and then incubated with normal goat serum for 1 h. Next, the sections were incubated with rabbit anti-EphA5 polyclonal antibody (dilution 1:1000, Abcam, CA) for 1 h at 37°C and then stained using an HRP-conjugated secondary antibody (Dako, UK) for 1 h at room temperature. Finally, the sections were incubated and stained with DAB substrate and hematoxylin, scanned with an Olympus BX53 microscope and photographed using the Cellsens Entry software (Olympus). EphA5 expression was classified as negative if less than 5% of the tumor cells were positive for EphA5 staining and classified as positive if more than 5% of the tumor cells were positive for EphA5 staining.

### Scratch migration assay and Invasion assay

For evaluation the EphA5 function,we obtained the Du145 derivative cell lines that stably overexpressed EphA5 via transfection of pCMV6-AC-GFP-EphA5 plasmid (Cat No. RG213206, Origene, CA) according to the manufacturer’s protocol. Du145 cell transfected with pCMV6-AC-GFP empty plasmid (Cat No. PS100010, Origene, CA) empty vector were used as the control.

For scratch migration assay, cells were cultured in 24-well plates until confluence. The monolayer was scratched with a sterile 200 μl pipette tip to create a denuded area of constant width. The wound closure was monitored and photographed before and 24 hours after wounding.

For the Matrigel invasion assay, 1 × 10^5^ cells were seeded into the upper compartment of the insert with Matrigel (BD Biosciences, Woburn, Mass) in serum-free growth medium. Then, the upper chamber were placed into 24-well culture dishes containing 600 μl of complete growth medium. After 48h of incubation at 37°C, cells in the upper chamber were subsequently removed with cotton swabs and then stained with a solution containing 0.1% crystal violet and 4% formaldehyde. The number of cells that fixed on the bottom membrane of the inserts was counted.

### Statistics

A two-tailed Student's *t*-test was used to compare various groups to assess statistical significance. The differences in gene expression levels between prostate cancer samples and noncancerous prostate tissue specimens were analyzed using a chi-squared test. All statistical analyses were performed using the SPSS 11.0 software. *P* < 0.05 was considered to be statistically significant.

## Results

### Down-regulation of EphA5 in prostate cancer

**Figure 1:**
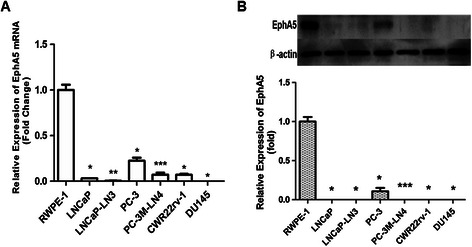
**qRT-PCR and Western blot analysis of EphA5 expression in prostate cell lines. A**, EphA5 mRNA expression was analyzed by qRT-PCR in 7 prostate cell lines. GAPDH was amplified as an internal control. **B**, Western blot analysis of EphA5 protein (114 kDa band) expression in 7 prostate cell lines. β-Actin (43 kDa band) was used as a control for equal loading of cell lysates. Representative results of triplicate experiments are shown as mean ± SD (n = 3). *P < 0.05, vs. the RWPE-1 cell line; **P < 0.05, vs. the LNCaP cell line, ***P < 0.05, vs. the PC-3 cell line.

To explore the potential role of EphA5 in prostate carcinogenesis, we first analyzed its expression by real-time PCR in a panel of human nonmalignant (RWPE-1) and prostate cancer (LNCaP, LNCaP-LN3, PC-3, PC-3M-LN4, CWR22rv-1, and DU145) cell lines. EphA5 mRNA expression was significantly decreased in all six prostate cancer cell lines compared to the nonmalignant RWPE-1 cells (Figure 1A). In addition, we also observed that EphA5 gene expression was decreased consistently and significantly in both lymph node derivative cell lines compared to their parental prostate cancer cells LNCaP and PC-3 (Figure 1A). We further investigated the EphA5 protein levels by Western blotting analyses in these cell lines. The protein levels were consistent with the respective mRNA levels for the various cell lines (Figure 1B).

To determine whether epigenetic silencing of the EphA5 gene also occurs in primary prostate tumors, EphA5 expression was analyzed by real-time PCR in 39 BPH tissues, 22 primary prostate tumor tissues and 23 paired noncancerous and tumor tissues. EphA5 mRNA expression was downregulated in 28 of 45 (62.2%) prostate cancer samples and in 2 of 39 (5.1%) BPH samples (Table 2). Among the 23 paired prostate carcinoma specimens, 15 (65.2%) tumor tissues exhibited the downregulation of EphA5 when compared with their respectively matched noncancerous tissues (Additional file 1: Table S1).

**Table 2:** **Correlation of EphA5 methylation and mRNA expression with clinical and histological parameters in PCa patients**

	Methylation		*p*-value^1^	mRNA expression		*p*-value^1^
	Present	Absent		Normal	Reduced	
Age (years)						
≤70	18	7	0.883	8	17	0.371
>70	14	6		9	11	
PSA (ng/ml)						
≤10	6	3	0.742	2	7	0.537
>10	26	10		15	21	
Stage (TNM)						
T1-T2	6	7	0.019	7	6	0.156
T3-T4	26	6		10	22	
Gleason score						
6-7	12	10	0.016	12	10	0.023
8-10	20	3		5	18	
Prostate volume (ml)						
≤50	24	7	0.165	11	20	0.637
>50	8	6		6	8	

Normal: 0.5 ≤ 2^-△△Ct^ ≤2; Reduced: 2^-△△Ct^ < 0.5; ^1^χ^2^ (2-tailed).

**Figure 2:**
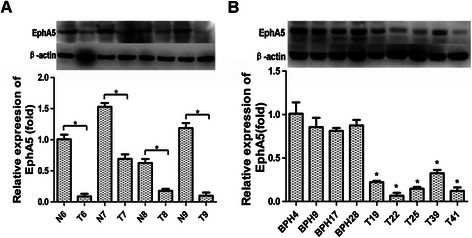
**Western blotting analysis of EphA5 protein expression in clinical prostate specimens. A**, EphA5 protein (114 kDa band) levels in representative resected fresh prostate cancer tissues and matched non-tumor tissues were analyzed by western blotting and normalized to β-actin expression (43 kDa band). Expression was further normalized to the expression level observed in the first noncancerous specimen. **B**, EphA5 protein levels (114 kDa band) in representative fresh prostate cancer tissues (collected via biopsy) and benign prostate hyperplasias were analyzed by western blotting and normalized to β-actin (43 kDa band). Protein levels were further normalized to the expression level observed in the first benign prostate hyperplasia specimen. Representative results from triplicate experiments are shown as mean ± SD (n = 3). *P < 0.05, vs. the respective noncancerous tissue and BPH. N = non-tumor tissue; T = tumor tissue; BPH = benign prostate hyperplasia.

To further validate the expression of EphA5 in human PCa tissue, we also analysed 4 BPH tissues, 5 primary prostate tumors tissues and 4 paired normal tissues in our study by Western blotting assay. Similar to the results of qRT–PCR in the corresponding tissues, EphA5 protein level in prostate tumour samples was significantly lower than that of matched adjacent normal tissues or BPH tissues (Figure 2).

### Methylation status of EphA5 in prostate cancer

**Figure 3:**
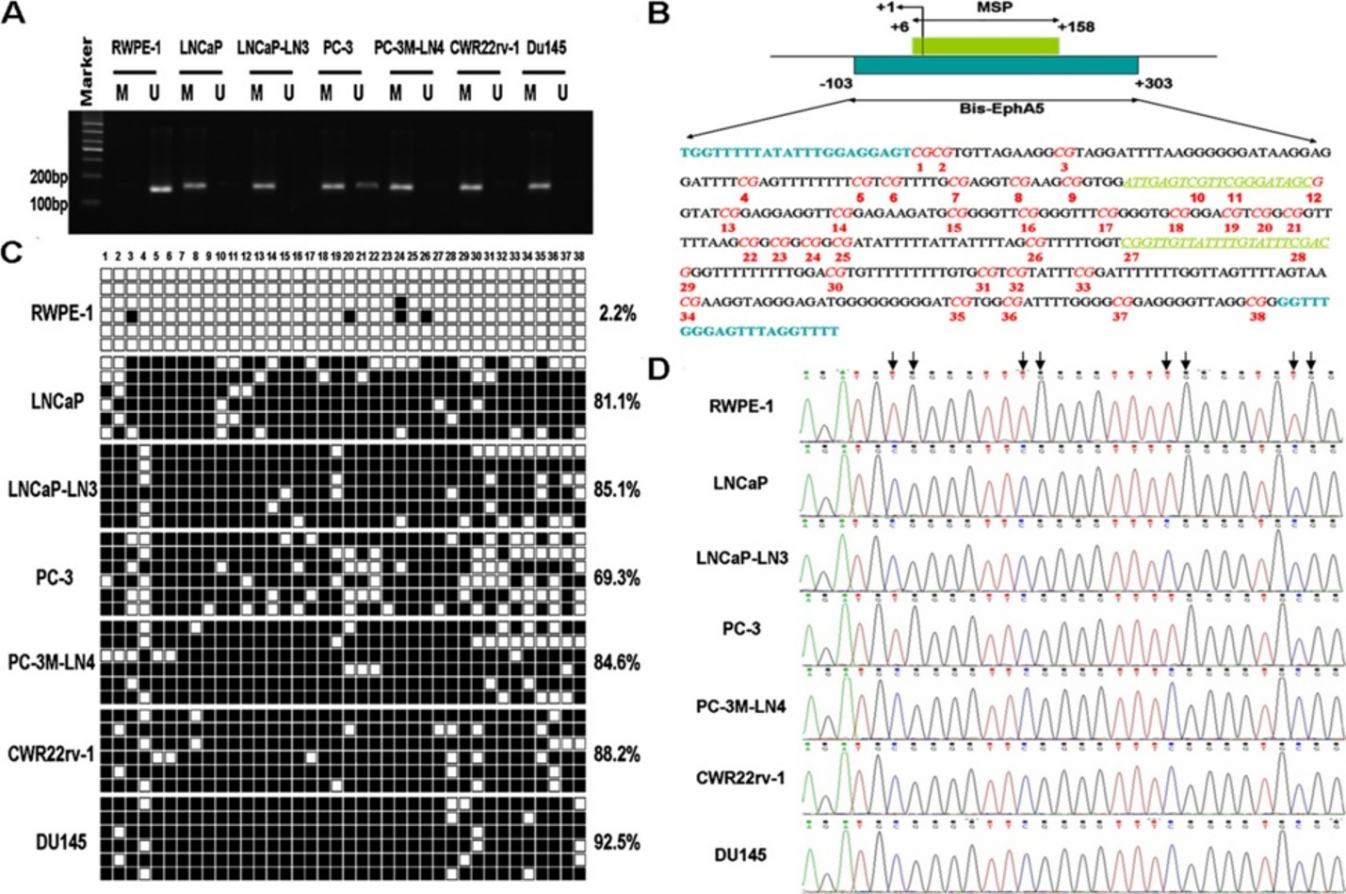
**Methylation status of the EphA5 gene promoter in prostate cell lines. A**, EphA5 methylation status was determined by MSP-PCR analysis. All prostate cancer cell lines exhibited complete methylation of the EphA5 gene. Unmethylated EphA5 alleles were detected in RWPE-1 and PC-3 cell lines. Lanes labeled “M” and “U” denote products amplified with primers recognizing methylated and unmethylated sequences, respectively. **B**, Schematic depiction of the EphA5 promoter-associated CpG island, which spans the region from -103 to +303 with respect to the TSS (+1). The bisulfite sequencing PCR primers are shown in light blue and bold type. The MSP = PCR primers are highlighted in khaki, italicized, and underlined. There are 38 CpG sites in this region; the CpG sites are numbered in red and bold type. **C**, Methylation patterns of individual EphA5 promoter clones from prostate cell lines that were sequenced using bisulfite methods. Six clones from each sample were bisulfite sequenced to obtain a representative sampling of methylation patterns; CpG dinucleotides are represented by squares (■, methylated cytokines; □, unmethylated cytosines). Cell-line names and the percentage of methylation for the corresponding cell line are indicated on the left and right sides, respectively. **D**, Representative chromatograms of CpG sites 14 to 18 obtained from bisulfite sequencing of the EphA5 fragment. Arrows indicate positions of CpG dinucleotides.

To determine the potential mechanism of EphA5 downregulation in prostate cancer, we analyzed the EphA5 gene 5′ regulatory region. We found a CpG island encompassing the transcription start site (TSS) of EphA5. Then, methylation-specific PCR (MSP-PCR) was performed to examine the methylation status of each of the cell lines. Methylated DNA was detected in all six prostate cancer cell lines, whereas the hypermethylation of EphA5 gene was not detected in nonmalignant RWPE-1 cells (Figure 3A). In addition, both methylated and unmethylated sequences were observed in the PC-3 cell line, indicating partial methylation.

**Figure 4:**
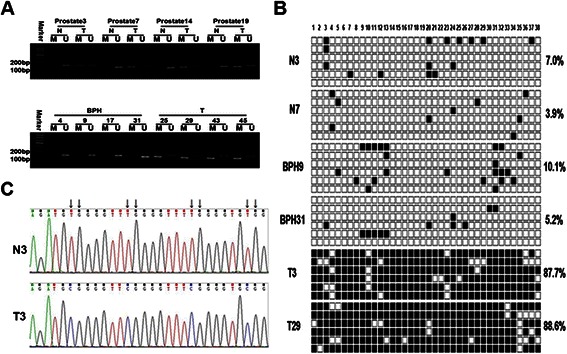
**Methylation status of the EphA5 gene promoter in prostate tissue. A**, EphA5 methylation status was determined by MSP-PCR. All of the prostate cancer tissues exhibit complete methylation of the EphA5 gene. The unmethylated alleles were detected in adjacent noncancerous (top) and BPH (bottom) tissues. Lanes labeled “M” and “U” denote products amplified by primers recognizing methylated and unmethylated sequences, respectively. **B**, Methylation patterns of individual EphA5 promoter clones from prostate tissue that were bisulfite sequenced are shown. Six clones from each sample were bisulfite sequenced to obtain a representative sampling of methylation patterns; CpG dinucleotides are represented by squares (■, methylated cytokines; □, unmethylated cytosines). Sample names and the methylation percentage of the corresponding tissue are indicated on the left and right sides, respectively. **C**, Representative examples of an unmethylated EphA5 CpG island in sample N1 (top) and a highly methylated CpG island in sample T24 are shown, as determined by bisulfite sequencing analysis. Arrows indicate positions of CpG dinucleotides.

To determine whether EphA5 hypermethylation also occurs in primary prostate tumors, the methylation status of EphA5 was determined by MSP-PCR in 39 BPH tissues, 22 primary prostate tumor tissues and 23 paired noncancerous and tumor tissues. The frequency of EphA5 promoter methylation was significantly higher in prostate cancer samples (32 of 45, 71.1%) than in BPH tissue samples (5 of 39, 12.8%; p < 0.01) and paired noncancerous tissues (2 of 23, 8.7%; p < 0.01). Of these 32 methylated prostate cancer samples, EphA5 expression was markedly downregulated in 25 samples. The correlation between EphA5 expression and hypermethylation of the CpG island was significant (p = 0.001). Among the 23 paired prostate carcinoma specimens, the hypermethylation of EphA5 was detected in 69.6% (16/23) prostate carcinoma tissues. Of these 16 prostate cancer samples, 15(93.8%) exhibited the downregulation of EphA5 expression than that of their respectively matched noncancerous tissues, implying that the hypermethylation of EphA5 was significantly correlated with the downregulation of EphA5 (*p* < 0.01) (Additional file 1: Table S1). The unmethylated form of EphA5, which was present in all samples, is likely due to the inherent contamination with normal (nonmalignant) cells or partial methylation. Representative results from MSP–PCR analyses in prostate tissue are shown in Figure 4A.

To further verify the MSP-PCR results, we subjected all of the cell lines and randomly selected tissue samples to bisulfite sequencing. We analyzed a 406 bp segment of the EphA5 gene 5′ regulatory region (−103 to +303 bp; TSS, +1 bp), which includes 38 CpG sites and spans the core promoter, exon 1, and part of intron 1 (Figure 3B). Similar to the MSP-PCR results, the CpG sites were hypermethylated in all six tumor cell lines (LNCaP, LNCap-LN3, PC-3, PC-3M-LN4, CWR22rv-1, and DU145), and in some of the prostate carcinoma and hyperplasia specimens (Figures 3C and 4B). Representative examples displaying frequent, localized methylation at the CpG island for all six prostate tumor cell lines and a prostate carcinoma sample (T24) are shown in Figure 3D. Additionally, the lack of methylation in the RWPE-1 nonmalignant cell line and a noncancerous prostate sample (N1) are shown in Figure 4C.

### Immunohistochemical expression of EphA5

**Figure 5:**
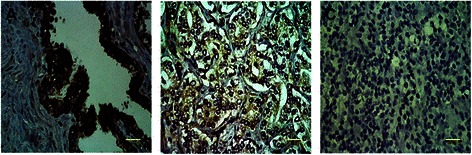
**Representatively immunostaining analysis of EphA5 in prostate tissue. A**. Strong cytoplasmic expression of EphA5 in the adjacent noncancerous prostate tissue sample N8. **B**. Weak cytoplasmic expression of EphA5 in the prostate carcinoma tissue sample T6 (Gleason score = 3 + 4). **C**. Complete loss of EphA5 expression in the prostate carcinoma sample T8 (Gleason score = 4 + 5) (scale bar = 50 μm).

To further verify the expression of EphA5 in human prostate tumors, we examined its expression via immunohistochemistry in 13 paired prostate carcinomas and noncancerous tissues. Strong immunostaining of the EphA5 protein was observed in the cytoplasm of all 13 paired noncancerous tissues. Among the 13 prostate carcinoma specimens, 10 (76.9%) exhibited undetectable or weak immunostaining. Of these 10 tumor tissues, hypermethylation was present in 8 samples (80.0%). There was a strongly negative correlation between promoter hypermethylation of the EphA5 gene and EphA5 protein expression (*p* = 0.012). Representative examples displaying positive EphA5 protein immunostaining in a noncancerous prostate sample, weak EphA5 protein immunostaining in a low-grade prostate cancer specimen (Gleason score = 7) and negative EphA5 protein immunostaining in a high-grade prostate carcinoma sample (Gleason score = 9) are shown in Figure 5.

### Restoration of EphA5 gene expression by treatment with 5-aza-2′-deoxycytidine

**Figure 6:**
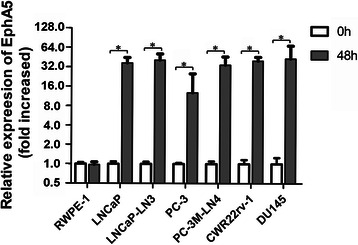
**EphA5 mRNA expression analysis by qRT-PCR in 7 prostate cells lines following 5-aza-2'-deoxycytidine (5 μmol/l) treatment for 0 and 48 hr.** EphA5 gene expression levels for cell lines treated with 5-aza-2'-deoxycytidine are shown as the relative fold change compared to untreated cells (0 hours, defined as 1.0). Representative results from experiments conducted in triplicate are shown as mean ± SD. *Denotes statistical significance at *P* < 0.05 compared to values at 0 hours.

To determine whether the inhibition of cytosine methylation could induce EphA5 mRNA expression in cell lines with hypermethylated CpG islands, we treated all of the cell lines with the cytosine methylation inhibitor 5-aza-2′-deoxycytidine (5 μmol/L, 48 h). Compared with untreated cells, the EphA5 mRNA level was significantly increased in LNCaP (36.7 ± 5.9-fold, *p* = 0.0021), LNCaP-LN3 (40.6 ± 4.4-fold, *p* = 0.0001), PC-3 (12.7 ± 3.7-fold, *p =* 0.0018), PC-3M-LN4 (33.6 ± 7.3-fold, *p* = 0.0041), CWR22rv-1 (39.1 ± 4.8-fold, *p* = 0.0063), and DU145 (42.9 ± 9.8-fold, *p* = 0.0039) cells. However, there was no significant enhancement of EphA5 mRNA levels in the RWPE-1 nonmalignant cells following 5-aza-2′-deoxycytidine treatment for 48 h (Figure 6). These results suggest that DNA hypermethylation is involved in EphA5 gene silencing in prostate cancer cell lines.

### Correlation between the mRNA expression and methylation of EphA5 and clinicopathologic features

We analyzed various patient clinicopathologic parameters in relation to the downregulation and methylation of EphA5. EphA5 expression differences were not correlated with median age (*p* = 0.371), TNM stage (*p* = 0.156), PSA serum concentration (*p* = 0.537) or prostate volume (*p* = 0.637). However, the low Gleason score group (Gleason score: 6-7) had significantly higher expression of EphA5 than the high Gleason score group (Gleason score: 8-10) (45.5% vs. 78.3%; *p* = 0.023). Significant correlations were observed between the frequency of EphA5 methylation and TNM staging (*p* = 0.019) and Gleason score (*p* = 0.016). However, no significant difference between the Gleason score 7 (3 + 4) group and the Gleason score 7 (4 + 3) group was observed about the expression of EphA5 and the hypermethylation of EphA5 (data not shown). No correlation between the occurrence of EphA5 hypermethylation and prostate-specific antigen (PSA) levels and prostate volume in prostate carcinoma samples was observed (Table 2).

### Overexpression of EphA5 inhibits prostate cancer cell invasion and migration

**Figure 7:**
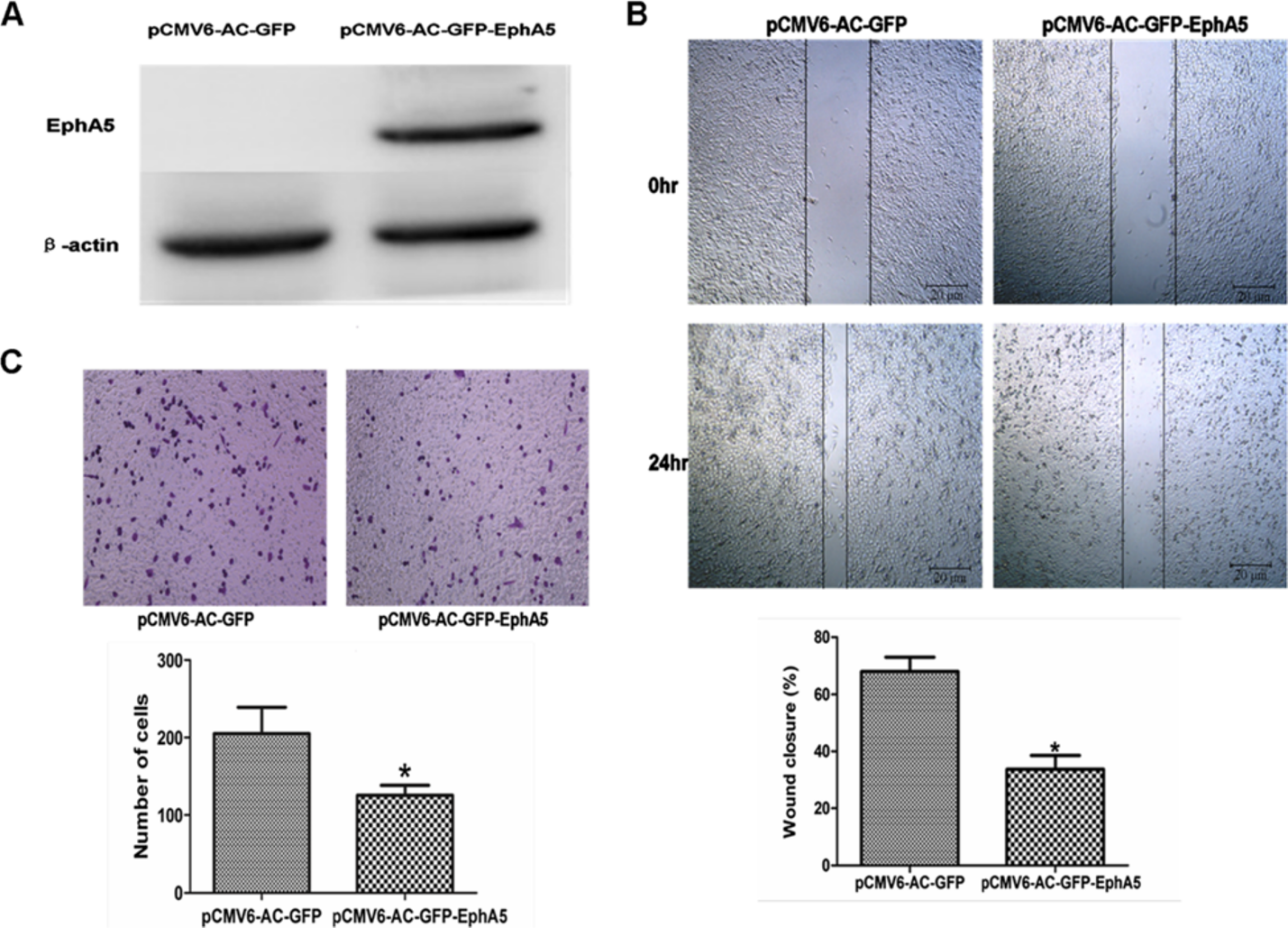
**EphA5 overexpression inhibited the migration and invasiveness of Du145 cells. A**. Western blot analysis of EphA5 protein (114 kDa band) expression in DU145 cells after transfection with pCMV6-GFP-EphA5. **B**. Cell migration activity determined with the wound healing assay. **C**. Cell invasion activity determined with the Matrigel invasion assay (×100). All experiments were performed in triplicate. Data are shown as the mean ± SD. *P < 0.05 vs. pCMV6-AC-GFP group.

To assess the effect of EphA5 expression on invasion and migration, we transiently infected Du145 cells lacking expression of EphA5 with plasmid expressing pCMV6-AC-GFP or pCMV6-AC-GFP-EphA5 and generated stably transfected cells using G418 selection. Western blotting analysis demonstrated that EphA5 was over-expressed in DU145 cells transfected with pCMV6-AC-GFP-EphA5 (Figure 7A). The wound healing assay demonstrated that the wound-closure rate of pCMV6-AC-GFP-EphA5 cells decreased by 33.7% (*p* <0.05) when compared with control pCMV6-AC-GFP cell (Figure 7C). The transwell assay showed that the number of invasive cells in pCMV6-AC-GFP-EphA5 cells decreased by 38.1% (*p* <0.05) when compared with control pCMV6-AC-GFP cell (Figure 7B). These data implied that EphA5 expression inhibited cell migration and invasion in vitro.

## Discussion

In this study, we systematically evaluated the expression profile and methylation status as well as its clinical relevance of the EphA5 gene in prostate cell lines, benign prostatic hyperplasia, primary human prostate tumors, and paired noncancerous tissues. The EphA5 gene was frequently silenced by an epigenetic alteration, namely DNA methylation in prostate cancer cell lines and tissues. To the best of our knowledge, the present study is the first to report the relationship between EphA5 expression level and its methylation status in prostate cancer.

Numerous reports have demonstrated that, similarly to the other members of the Eph family, EphA5 not only is involved in a variety of developmental processes [14,17,18], but also plays important roles in carcinogenesis and the tumor progression of many cancers [10,15,19-23]. A recent report revealed that EphA5 expression was decreased in low-grade glioma tumor tissues and was further reduced in high-grade glioma tumor tissues compared to normal control brain tissues, which suggested a novel role of EphA5 as a tumor suppressor [19]. Moreover, the downregulation of EphA5 expression was also observed in other advanced tumors, including colorectal cancer, acute lymphocytic leukemia, and breast cancer [10,15,20,21]. Consistent with previous studies, the downregulation of EphA5 expression was also observed in 28 of 45 (62.2%) prostate cancer tissues with various histological stages and in all 6 PCa cell lines. The downregulation of EphA5 was also associated with increased Gleason score in prostate cancer. Another fact that supports the importance of EphA5 in prostate cancer metastasis is that EphA5 transcript and protein levels were reduced consistently and significantly in both of the lymph node-derived cell lines compared with their parental prostate cancer cells (LNCaP and PC-3). The evidence suggests a potentially suppressive role of EphA5 transcripts in prostate carcinoma progression.

DNA methylation/demethylation at promoter cytosine residues within conserved CpG islands is a powerful epigenetic modification that regulates gene transcription, and aberrant methylation can lead to gene silencing of critical genes and has been a well-documented phenomenon in many malignancies, particularly in prostate carcinoma [8-11,20,24]. Recently several groups demonstrated EphA5 promoter methylation in breast cancer, colorectal cancer and acute lymphoblastic leukemia [10,15,20], implying that the hypermethylation of EphA5 paly an important role in cancer progress. Similarly to previous studies, EphA5 gene hypermethylation was also detected in all 6 PCa cell lines and in 71.1% of tumor samples, whereas only 12.8% of benign prostatic hyperplasia and 8.7% of paired noncancerous tissues exhibited hypermethylation of the EphA5 gene within the same CpG islands as the study on breast tumor. These findings suggest that EphA5 hypermethylation occurred specifically during prostate tumorigenesis and indicate that EphA5 could be used as a potential marker to distinguish malignant prostate tissue from nonmalignant tissue. Moreover, the significant associations of EphA5 methylation with higher clinicopathologic TNM staging and Gleason score support the important role of EphA5 in cancer progression. TNM staging and Gleason score are two practical parameters that are often used to estimate prostate cancer prognosis. Therefore, EphA5 methylation might be useful as a marker for biological aggressiveness and as a valuable prognostic predictor of prostate cancer.

Additionally, to elucidate the mechanisms leading to the downregulation of EphA5 expression in prostate cancer, we determined the relationship between transcript expression level and the methylation status of EphA5 in prostate cancer cell lines and tissues. A significant correlation was observed between the downregulation of EphA5 expression and EphA5 methylation in prostate cancer. EphA5 expression was downregulated in all 6 PCa cell lines harboring the methyaltion. Furthermore, the downregulation of EphA5 expression could be reversed in all 6 PCa cell lines by treating cells with the DNA demethylating agent 5-aza-2′-deoxycytidine, implying that hypermethylation was an important reason for promoting the downregulation of EphA5 expression in prostate cancer. Our findings also provided a good explanation for a previous study that EphA5 mRNA levels were decreased in high-grade (Gleason score = 8) PCa tissues compared to low-grade (Gleason score = 6) PCa tissues [16].

To further evaluate whether EphA5 played a functional role in the PCa, we assessed the changes of biological characteristics in PCa cell line (Du145) after ectopic expression of EphA5. We found that EphA5 overexpression significantly decreased Du145 cell migratory and invasive capabilitie in vitro, suggestting that EphA5 may potentially suppress prostate cancer metastasis.

Paradoxically, recent studies [22,23] have demonstrated that the EphA5 gene was upregulated in high-grade hepatocellular carcinoma. The findings described above prompted us to ask whether EphA5 receptor can act in a bimodal manner. Similar questions have already been addressed for other Eph receptors, such as EphA2, EphA7, and EphB4 [9,25-28]. These findings could also be reflective of Eph/ephrin functions that are influenced by tissue type, oncogenic context, or ligand-independent versus ligand-dependent signaling. Therefore, the detailed role of EphA5 in prostate cancer warrant further investigation.

## Conclusion

In summary, we have established that loss of EphA5 expression in prostate cancer may be due to methylation of CpG sites within the EphA5 promoter. Our data indicate that EphA5 is a potential prognostic biomarker and a useful molecular therapeutic target to attenuate prostate cancer progression.

## Additional file

Additional file 1: Table S1. Correlation between EphA5 expression and methylation in 23 paried prostate tumor tissues.
