# Supplementary material for: Downregulation of EphA5 by promoter methylation in human prostate cancer
Source: BMC Cancer. 2015 Jan 22;15:18. doi: 10.1186/s12885-015-1025-3 (PMC4307617; doi:10.1186/s12885-015-1025-3)
Supplement: Additional file 1: Table S1. — Correlation between EphA5 expression and methylation in 23 paried prostate tumor tissues. [file 12885_2015_1025_MOESM1_ESM.doc]

**Supplementary Table I . Correlation between EphA5 expression and methylation in 23 paried prostate tumor tissues**

| mRNA expression | Methylation（MSP） | | p-Value^1^ |
| --- | --- | --- | --- |
|  | Present（n=16） | Absent（n=7） |  |
| Normal（n=8） | 1 | 7 | ＜0.001 |
| Reduced（n=15） | 15 | 0 |  |

Normal: 0.5≤2^-△△Ct^ ≤2; Reduced：2^-△△Ct^<0.5; ^1^χ^2^(2-tailed).
